# Friction Behaviour of 6082-T6 Aluminium Alloy Sheets in a Strip Draw Tribological Test

**DOI:** 10.3390/ma16062338

**Published:** 2023-03-14

**Authors:** Tomasz Trzepieciński, Ján Slota, Ľuboš Kaščák, Ivan Gajdoš, Marek Vojtko

**Affiliations:** 1 Department of Manufacturing Processes and Production Engineering, Rzeszow University of Technology, al. Powst. Warszawy 8, 35-959 Rzeszów, Poland; 2 Institute of Technology and Material Engineering, Faculty of Mechanical Engineering, Technical University of Košice, Mäsiarska 74, 040 01 Košice, Slovakia; 3 The Institute of Materials Research, Slovak Academy of Sciences, Watsonova 47, 040 01 Košice, Slovakia

**Keywords:** 6082-T6, aluminium alloy, coefficient of friction, sheet metal forming, surface topography, ANN

## Abstract

Aluminium alloy sheets cause many problems in sheet metal forming processes owing to their tendency to gall the surface of the tool. The paper presents a method for the determination of the kinematic friction coefficient of friction pairs. The determination of coefficient of friction (COF) in sheet metal forming requires specialised devices that ‘simulate’ friction conditions in specific areas of the formed sheet. In this article, the friction behaviour of aluminium alloy sheets was determined using the strip drawing test. The 1-mm-thick 6082 aluminium alloy sheets in T6 temper were used as test material. Different values for nominal pressures (4.38, 6.53, 8.13, 9.47, 10.63, and 11.69 MPa) and different sliding speeds (10 and 20 mm/min.) were considered. The change of friction conditions was also realised with several typical oils (hydraulic oil LHL 32, machine oil LAN 46 and engine oil SAE 5W-40 C3) commonly used in sheet metal forming operations. Friction tests were conducted at room temperature (24 °C). The main tribological mechanisms accompanying friction (adhesion, flattening, ploughing) were identified using a scanning electron microscope (SEM). The influence of the parameters of the friction process on the value of the COF was determined using artificial neural networks. The lowest value of the COF was recorded when lubricating the sheet metal surface with SAE 5W40 C3 engine oil, which is characterised as the most viscous of all tested lubricants. In dry friction conditions, a decreasing trend of the COF with increasing contact pressure was observed. In the whole range of applied contact pressures (4.38–11.69 MPa), the value of the COF during lubrication with SAE 5W40 C3 engine oil was between 0.14 and 0.17 for a sliding speed of 10 mm/min and between 0.13 and 0.16 for a sliding speed of 20 mm/min. The value of the COF during dry friction was between 0.23 and 0.28 for a sliding speed of 10 mm/min and between 0.22 and 0.26 for a sliding speed of 20 mm/min. SEM micrographs revealed that the main friction mechanism of 6082-T6 aluminium alloys sheet in contact with cold-work tool steel flattens surface asperities. The sensitivity analysis of the input parameters on the value of COF revealed that oil viscosity has the greatest impact on the value of the COF, followed by contact pressure and sliding speed.

## 1. Introduction

Sheets made of aluminium and aluminium alloys, due to their favourable ratio of strength to weight, are being used increasingly often in the automotive industry. Adding alloying elements to aluminium can increase its strength properties several times [1]. Alloys obtained in this way are characterised by low density and high impact strength. Nickel and cobalt, as well as magnesium and manganese, increase their ‘strength’ properties, and titanium and chromium affect grain size reduction [2]. Wrought alloys typically contain up to 5% alloying elements and are used in a hardened and heat-treated condition. In special conditions, cast aluminium alloys can also be processed by plastic working [3]. However, aluminium-based alloys generally have relatively low fatigue strength [4]. The fatigue resistance of aluminium alloys can be improved by adding elements belonging to the group of transition metals, including titanium, vanadium, and zirconium [5]. In this paper, the frictional properties of AW-6082-T6 aluminium alloy sheets are presented. The 6xxx series alloys contain magnesium (0.2–3%) and silicon (0.2–1.8%) as the main alloying additions. Some of the 6xxx series alloys contain manganese (up to 1.4%) and copper (up to 1.2%). Alloys in this group show good formability and are susceptible to machining. Those that do not contain copper have good corrosion resistance and can be anodized. Typical applications of aluminium alloys containing magnesium and silicon include structural elements of motor vehicles, interior fittings, and profiles for the construction industry.

A phenomenon accompanying friction in the formation of aluminium and aluminium alloy sheets is the galling of the surface of tools with the sheet material. In this way, the changing topography of the tool surface causes an unfavourable change in the topography of the formed drawpiece and more resistance to the movement of the sheet metal on the surface of the tool [6]. Surface topography has a decisive influence on friction, wear, and lubrication under mixed lubrication and dry friction conditions [7,8]. In the case of forming sheets made of aluminium, adhesive wear is also of great importance, consisting of metallic local adhesion of the roughness asperities in the micro-areas of plastic deformation. The mechanism of coexistence of abrasive and adhesive wear may result in the extension of frictional contact time, manifesting itself in the intensification of the surface galling [9].

A common way to reduce the coefficient of friction in sheet metal forming is to use lubricants. The purpose of using lubricant is also to reduce the wear intensity of the friction pair elements [6,7]. The lubricant should meet a number of the following requirements, including being easy to apply to the metal being processed and the tool, having high resistance to normal loads, and being easily remove from the surface of the product [8,9]. The grease should be characterized by appropriate viscosity and chemical activity, which is the ability to form a protective layer on the friction surface [10]. Appropriate chemical activity of lubricants is provided by surface-active compounds, such as fatty acids (oleic, stearic, palmitic) and their salts. Different conditions for the implementation of sheet metal forming processes and the variety of plastically processed materials mean that the following parameters should be taken into account when selecting a lubricant for a specific application [11]: processing temperature, value of maximum unit pressures, grade of processed material and grade of tool material, sliding speed, type of protective coating on the tool surface, and tool design. The surface topography of sheet metal has a decisive influence on friction, wear, and lubrication under mixed lubrication and dry friction conditions [12,13].

The basic criteria for the division of lubricants are the consistency of the lubricant, origin (mineral or organic), and intended use [14]. Due to the consistency, lubricants are distinguished in the following groups: liquid lubricants (oils), emulsions (oil mists), and solid lubricants. Lubricating oils are obtained by mixing base oils with enriching additives [15,16].

Mineral oils are obtained by processing base oils, and synthetic oils are produced by chemical synthesis or the processing of mineral oils [17]. Mineral oils are complex mixtures of saturated and aromatic hydrocarbons with a ring or chain structure, containing 20 to 40 carbon atoms in a molecule [18]. Oils resulting from distillation from crude oil differ in viscosity, chemical composition, and physical properties. Due to their viscosity, mineral oils are divided into spindle, machine, motor, and gear oils.

In order to determine the friction and wear of materials, the following types of tests and tribometers may be used: (a) strip drawing test with flat [19,20] and (b) rounded [21] (c) countersamples, (d) bending-under-tension test [22,23], (e) draw-bead test [24,25], and (f) ball-on-disc [26], (g) block-on-disc [27], (h) pin-on-ring [28], and (i) pin-on-disc tribometers [29,30]. Most of the studies, the results of which can be found in the literature, indicate the susceptibility of aluminium sheets to galling and intensification of the flattening mechanism. As analysis of the literature showed, each aluminium alloy has a specific friction performance. Moreover, owing to poor formability of aluminium and aluminium alloys at room temperature, friction tests of alloys focus on plastic working in warm and cold forming conditions. Yahaya and Samion [31] analysed the friction condition of AW-6061 aluminium alloy lubricated with bio-lubricant in a cold forging test. It was found that the palm oil-based lubricant has good performance compared to a mineral-based oil in terms of surface roughness. However, the mineral oil had better friction performance than the palm oil-based lubricant. Trzepieciński [32] investigated the frictional performance of AW-2024-T3 Alclad aluminium alloy sheets using strip drawing test. Analysis of the effect of the friction conditions on the effectiveness of lubrication and change in the surface roughness of the metal sheets were analysed using analysis of variance (ANOVA). The other most commonly used friction tester is the pin-on-disc tribometer. Guezmil et al. [33] investigated the tribological behaviour of anodic oxide layer formed on AW-5754 aluminium alloy in a pin-on-disc tribometer. Different sliding speeds, normal loads, and oxide thicknesses were considered to establish the COF. It was found that increased normal load and sliding speed increased the COF. The effects of initial lubricant volume, temperature, sliding speed and contact pressure on the evolutions of the COF of AW-7075 aluminium alloy and the breakdown phenomenon were investigated by Yang et al. [34] in pin-on-disc tests. It was found that the COF rapidly increased as the lubricant film thickness decreased to a critical value. Das [35] studied the tribological properties of three aluminium as-cast alloy samples, i.e., Al-14 wt%, Al-10 wt%Si, and Al-7 wt%Si aluminium alloys, using a pin-on-disc type wear-testing machine. The wear of aluminium specimens was seen to increase at higher sliding speeds and at higher applied loads. Friction and wear on aluminium–silicon alloys have been extensively tested by Shabel et al. [36]. They identified two major types of wear relevant to industrial applications of Al–Si alloys: sliding wear and abrasive wear depending on the silicon particles, intermetallic constituents, and matrix hardness. Luis Pérez et al. [37] studied the friction properties of AW-5083 and AW-5754 aluminium alloys processed by equal channel angular pressing (ECAP). It was found that both nanostructured aluminium alloys show better wear behaviour if they are compared with conventional isothermal forging. Li et al. [38] also confirmed that ECAP processing leads to a decrease in the COF owing to improved mechanical properties. 

Many published works are focused on the analysis of the specific parameter of the friction process on the coefficient of friction or the occurrence of a specific friction mechanism. A considerable amount of factors in the friction process exist that affect the COF value and, as a result, building analytical friction model for specified process conditions is practically impossible. The artificial neural networks (ANNs) allow the researchers to overcome the difficulty arising in the assessment of the complex relationships between friction process parameters and COF. ANNs require a set of experimental training data to work properly. Based on the training process, ANNs acquire the ability to predict the value of the output parameter.

To the best knowledge of the authors, sheet metals made of AW-6082-T6 aluminium alloy have not been tested in strip drawing tests for pressures occurring in the blank holder zone during cold sheet metal forming. The selection of the appropriate lubricant is crucial to ensure appropriate conditions for the sheet metal forming of the automotive components.

Therefore, this article contains the results of the friction of AW-6082-T6 sheet metals. The friction behaviour of AW-6082-T6 aluminium alloy sheets was determined using the strip drawing test. Different values for nominal pressures and different sliding speeds were considered. The change of friction conditions was also realised with three typical lubricants (machine oil, engine oil and hydraulic oil) commonly used in sheet metal forming operations. The experimental design consists of 48 trials performed for two speeds, six pressures and four friction conditions. The tests were repeated three times to determine the average value of the coefficient of friction. The main tribological mechanisms accompanying friction were identified using a scanning electron microscope. Owing to the difficulty in determining the impact of the simultaneous interaction of many friction parameters on the value of the COF, artificial neural networks (ANNs) were used to identify the main relationships between the COF and process parameters. Sliding speed, average unit pressure, and lubricant viscosity were selected as input parameters of multilayer neural network. The output parameter was the value of the COF. The training of the network was carried out using the backpropagation algorithm.

## 2. Materials and Methods

### 2.1. Materials

The 1-mm-thick AW-6082 aluminium alloy sheets in T6 temper condition were used as test material. T6 indicates that the alloy has been solution heat-treated and, without any significant cold working, artificially aged to achieve precipitation hardening. The results of the friction tests will be used by the authors for the technological design of the forming process of automotive parts made of 6082-T6 aluminium alloy.

6082-T6 aluminium alloy is a wrought aluminium–magnesium–silicon family medium strength, weldable alloy with excellent corrosion resistance. The strength of this kind of this grade of aluminium alloy is the highest among all the alloys of the 6xxx series. 6082-T6 alloy is used for highly stressed applications in transport and marine frames.

**Figure 1:**
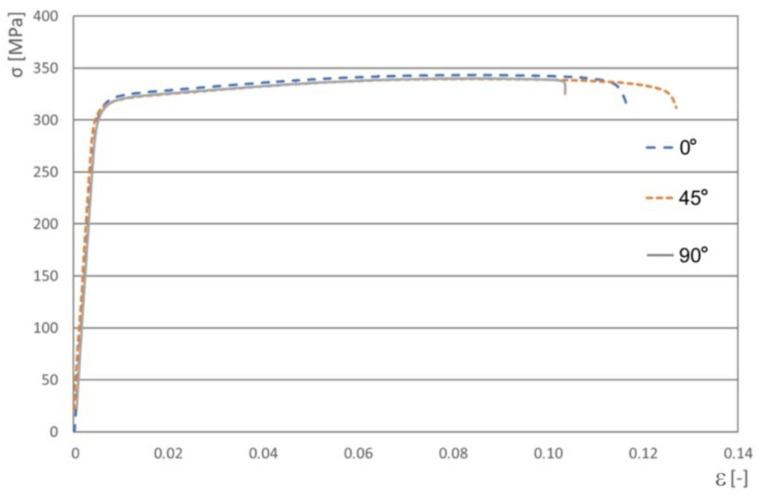
Engineering stress–strain curves for the EN AW-6082-T6 aluminium alloy sheets.

The basic mechanical parameters of the tested sheets (Table 1) were determined in a uniaxial tensile test according to the ISO 6892-1:2009 [39] on specimens that were cut transverse to the rolling direction (90°), along a rolling direction (0°), and at an angle of 45° according to the rolling direction of the sheet metal. Three specimens were tested for each direction and average values of mechanical parameters were determined. Engineering stress–strain curves for the characteristic sample directions are shown in Figure 1.

### 2.2. Friction Testing Procedure

**Figure 2:**
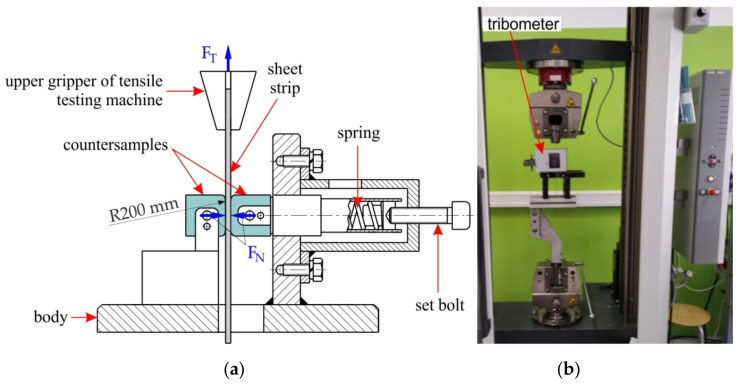
(**a**) Scheme and (**b**) view of tribological simulator.

Friction tests were carried out using the device (Figure 2) mounted on a Zwick/Roell Z100 testing machine. The test involves pulling a sample in the form of a strip of sheet metal with a width of w = 18 mm and a length of l = 240 mm clamped between cylindrical counter samples. Sliding speeds were 10 mm/min and 20 mm/min.

The friction force F_T_ is measured using the measuring system of the testing machine. During the test, the counterexamples were pressed against the strip sheet with the force F_N_, using a spring with a known deflection–axial force characteristic. Based on the values of the forces F_T_ and F_N_, the value of the kinematic coefficient of friction is determined, according to the relationship:

(1) $$\mu =\frac{F_{T}}{2F_{N}}$$

Counter samples made of 145Cr6 cold-work steel were used in the tests. To determine the mean contact pressure in the strip drawing test, the formulae proposed by Haar [40] (Equation (2)) was used, which was based on the width of the sample w, the contact force F_N_, the radius of the counter samples R = 200 mm, and the elastic properties of the sheet and counter-sample materials allow for determination of mean contact pressure p_av_.

(2) $$p_{\mathrm{av}}=\frac{\pi }{4}\cdot \sqrt{\frac{\frac{F_{N}}{w}\cdot \frac{2E_{1}E_{2}}{E_{2}\cdot \left(1-\nu_{1}^{2}\right)+E_{1}\cdot \left(1-\nu_{2}^{2}\right)}}{2\pi R}}$$

For the material of the steel counter sample, the following values of Young’s moduli E_1_ and Poisson’s ratio ν_1_ as E_1_ = 2·10^5^ GPa [41], ν_1_ = 0.3 [42] were adopted. The values of the same material parameters for the sample material were assumed as follows: E_2_ = 69,000 MPa [42], and ν_2_ = 0.33 [42].

The values of the applied pressure forces were between p_av_ = 4.38 MPa and 11.69 MPa, which, according to the literature [43,44,45,46], corresponds with the values of pressures occurring in the sheet metal forming operations.

All samples were degreased with acetone before the friction process. The friction tests were carried out in conditions of dry friction and sheet metal surface lubrication, with oils typically used in sheet metal forming. The basic criterion for the selection of lubricants was a wide range of viscosity η variability, which is the basic property of oils used in metal forming. In this way, three oils were selected:

- hydraulic oil LHL 32 (η = 21.9 mm^2^/s),
- machine oil LAN 46 (η = 43.9 mm^2^/s),
- and engine oil SAE 5W-40 C3 (η = 81 mm^2^/s).

The lubricant was applied directly to the surface of the samples.

**Figure 3:**
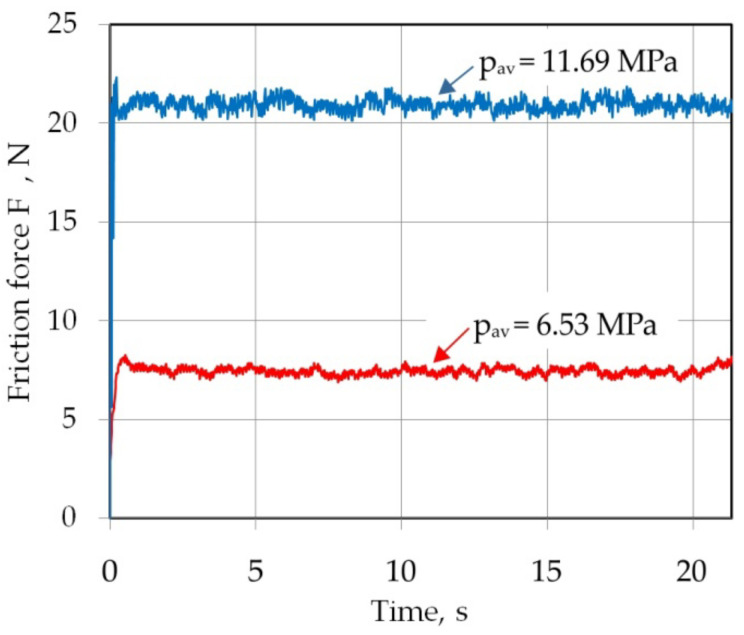
Example of force vs. time plots obtained in a friction test (dry friction conditions).

Experimental design of the friction tests is shown in Table 2. Three specimens were tested for each friction test and average values of the COFs have been determined. Some force vs. time plots are shown in Figure 3.

**Figure 4:**
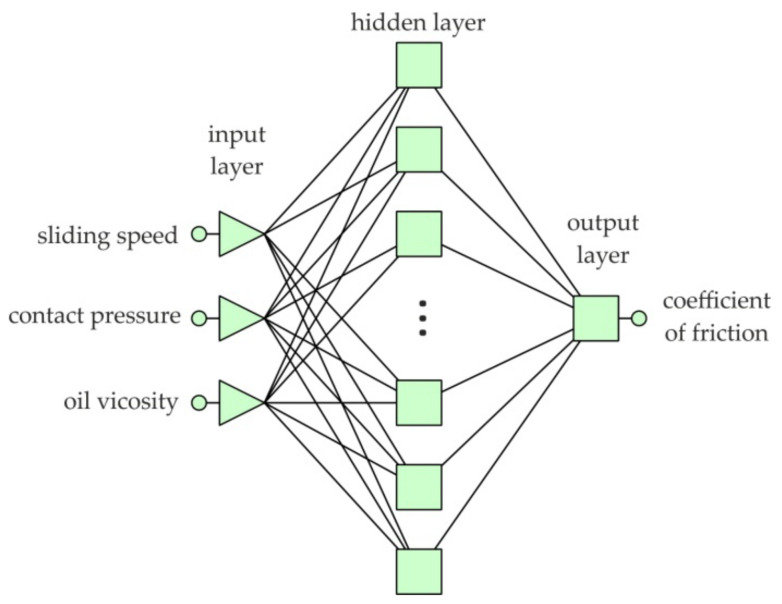
Structure of multilayer perceptron (MLP).

A T8000-RC surface measuring station was used to characterise the surface roughness of the sheet metals in their as-received state. The surface topography and basic 3D roughness parameters of sheet metal and counterexamples are shown in Figure 3 and Figure 4, respectively. 

### 2.3. Artificial Neural Networks

Designing a neural network using Statistica software includes several stages. First, a set of training data consisting of the input parameters and the corresponding value of the output parameter should be selected. Sliding speed, average unit pressure, and lubricant viscosity were selected as input parameters. The output parameter was the value of the coefficient of friction. The values of these parameters have been normalised to the range −1 to 1 [47], using min-max normalisation, which transforms the input data from the range (min, max) to the new range (N_min_, N_max_).

Many experiments were then carried out with selected multilayer network structures (Figure 4) in order to obtain the network with the smallest error for the validation set. The training of the network was carried out using the backpropagation algorithm which is commonly used to train multilayer neural networks. From the entire training data set, 15% of the data were selected and assigned to the validation set. The rest of the data comprised the training set. In general, the validation set should consist of between 10 and 20% of the training data. We have assumed that 15% of the data are summed to the validation set. The data of the validation set were used for independent convergence control of the training algorithm.

The quality of the tested neural networks was assessed on the basis of the root mean square (RMS) error.

The network with the smallest RMS error value for the validation set was used for data analysis. The selection of variables affecting the value of the coefficient of friction is difficult due to the synergistic interactions of many parameters often correlated with each other. The sensitivity analysis of the input variables showed a significant impact of all assumed input parameters on the value of the coefficient of friction.

## 3. Results and Discussion

### 3.1. Coefficient of Friction

**Figure 5:**
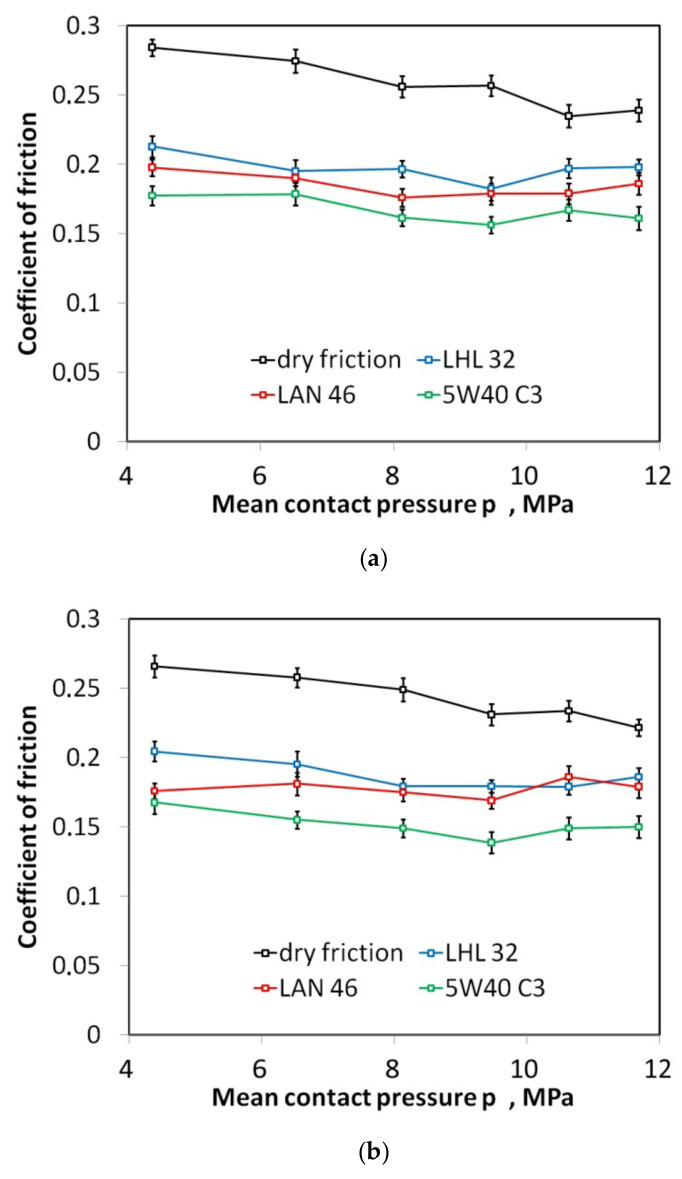
The effect of contact pressure on the coefficient of friction determined at sliding speed (**a**) 10 mm/min and (**b**) 20 mm/min.

The lowest value of the COF at room temperature (24 °C) was recorded when lubricating the sheet metal surface with SAE 5W40 C3 engine oil. In the whole range of applied contact pressures, the value of the COF during lubrication with this oil was between 0.15 (±0.006) and 0.18 (±0.008) for a sliding speed of 10 mm/min (Figure 5a) and between 0.14 (±0.0078) and 0.17 (±0.0083) for a sliding speed of 20 mm/min (Figure 5b). When lubricated with LAN 46 and SAE oils, an increase in the sliding speed from 10 to 20 mm/min resulted in a reduction of the COF by a maximum of 0.02. Based on the slope of the trend line of changes in the COF with the value of pressure, these lubricants are seen to provide lubrication to a similar degree for each pressure. After exceeding a pressure of 10 MPa, LAN 46 and LHL 32 oils clearly lose their lubricating properties. Under certain conditions, depending on the roughness of the cooperating bodies and the pressure value, the lubricating film breaks. It relates to an intensification of the mechanical cooperation of the roughness summits of the tool and the sheet metal. The results of friction tests of AW-5052 aluminium alloy sheets carried out by Dou and Xia [48] also showed that the values of the COF between the sheet metal and the die generally decrease with increasing sliding speed and normal loads, and the downward trend is slowed down with higher sliding speeds and contact pressure.

To determine the effectiveness of the lubricant in reducing the value of the coefficient of friction, the coefficient of effectiveness of lubrication (EOL) was introduced:

(3) $$\mathrm{EOL}=100\%-\frac{\mu_{\mathrm{dry}\;\mathrm{friction}}\cdot 100\%}{\mu_{\mathrm{lubrication}}}$$

**Figure 6:**
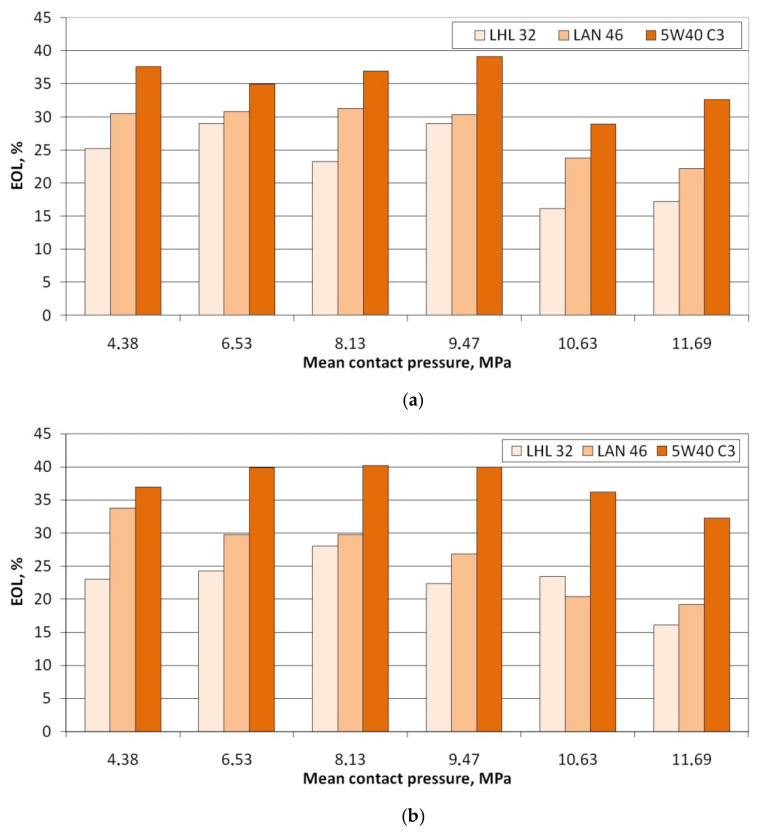
The effect of contact pressure on the EOL for sliding speed. (**a**) 10 mm/min and (**b**) 20 mm/min.

**Figure 7:**
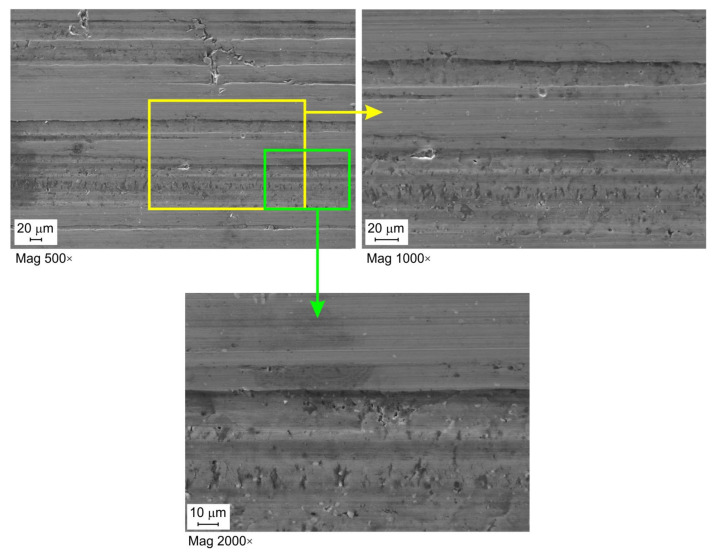
SEM micrographs of the sheet surface tested under dry friction conditions at contact pressure 8.13 MPa and a sliding speed of 10 mm/min.

Lubrication efficiency is the highest for oil with the highest viscosity 5W40 C3 (Figure 5a,b). For this oil, however, there is a tendency to initial stabilisation of lubrication efficiency at the pressures lower than 10 MPa, and then, after exceeding this pressure, the efficiency decreases. Despite the presence of increased lubricant pressure in the contact zone, the metallic contact is intensified, and the lubricant cannot separate rubbing surfaces very much under high contact pressure. The effectiveness of the other two oils (the LHL 32 oil provides the lowest lubrication efficiency) decreases practically over the entire range of applied contact pressures. In general, the value of the EOL coefficient for a specific pressure value increases with the increase in the sliding speed. The lubrication efficiency of LHL 32, LAN 46, and 5W40 C3 oils at a sliding speed of 10 mm/min (Figure 6a) ranges between 16.0 and 28.9%, 22.1 and 31.2%, and 28.9 and 39.1%, respectively. Meanwhile, at a sliding speed of 20 mm/min. (Figure 6b), the EOL values vary between 16.0 and 28.0%, 19.2 and 33.8%, and 32.3 and 40.2%, respectively. The increase in speed causes an increase in the temperature in the area of the summits of the surface asperities and thus an increase in their plastic properties [49]. The surface asperities are then more susceptible to plastic deformation at a lower value of pressure, causing faster flattening of the sheet surface (Figure 7) and reducing the volume of the lubricant pockets [50]. Liquid lubricants can reduce flattening and friction by filling the surface valleys and carry a substantial amount of the pressure [51].

Aluminium is easily oxidised in the air, so that in the initial period of friction, the oxide film easily separates the two surfaces of the material and there is a slight metallic contact transmitted through the surface asperities [52,53]. The oxide film has low shear strength and breaks quickly. After the load is applied, the top sheet layer cracks and the surfaces come into contact, which increases the bonding strength between the contacting surfaces [49]. Under lubricated conditions, with increasing contact pressure, the synergistic effect of the mechanisms of flattening and ploughing, and the action of the lubricant occurs. After a certain contact pressure is received, the increase in surface roughness and other parameters may reach a steady state value. Therefore, the values of the COF remain constant for the increased contact pressures [49]. This is visible in Figure 5 after exceeding the normal pressure value of 9 MPa.

**Figure 8:**
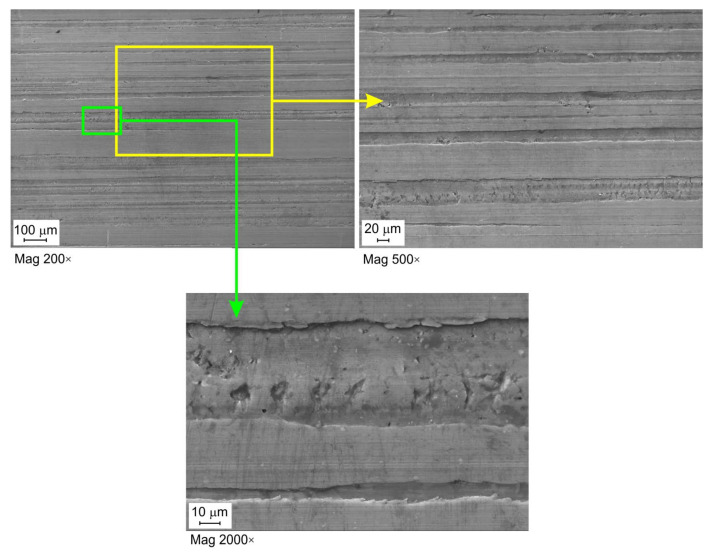
SEM micrographs of the sheet surface tested under lubrication with LHL 32 oil at contact pressure 9.47 MPa and sliding speed 10 mm/min.

**Figure 9:**
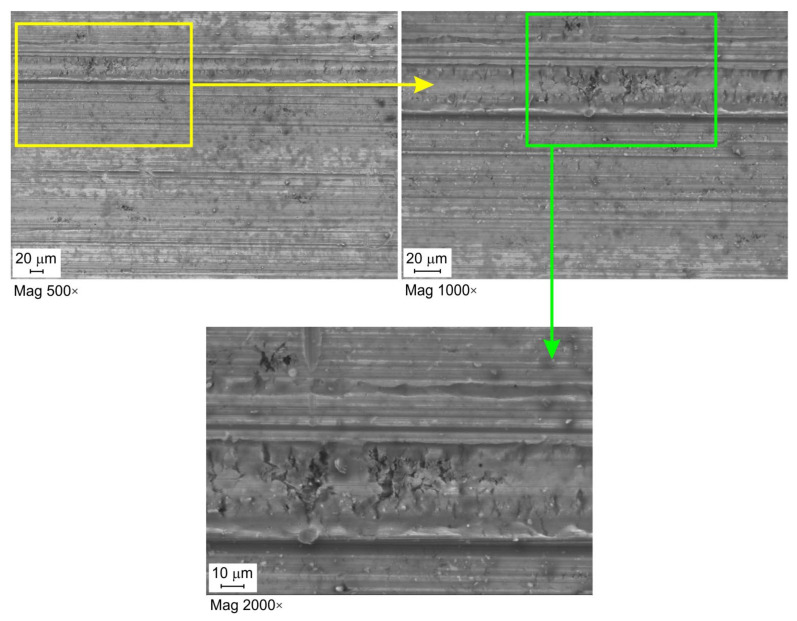
SEM micrographs of the sheet surface tested under lubrication with 5W40 C3 oil at contact pressure 6.53 MPa and sliding speed 10 mm/min.

**Figure 10:**
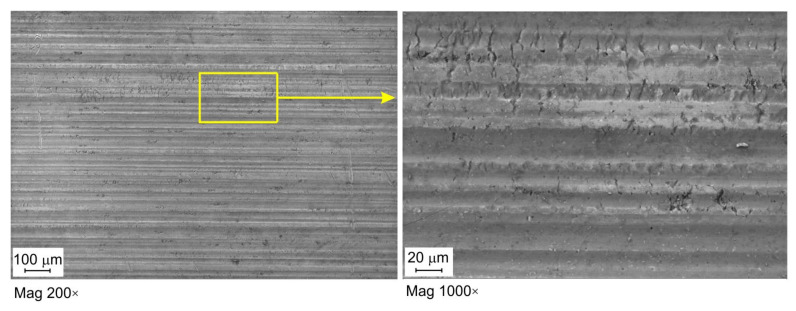
SEM micrograph of the as-received AW-6068-T6 sheet metal.

**Figure 11:**
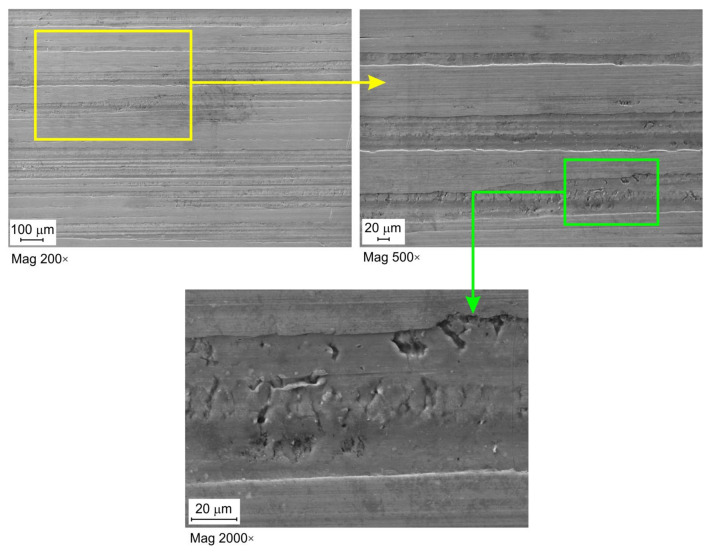
SEM micrographs of the sheet surface tested under lubrication with 5W40 C3 oil at contact pressure 10.63 MPa and sliding speed 20 mm/min.

Figure 7, Figure 8 and Figure 9 show selected surface morphologies of the surface of sheets tested at a sliding speed of 10 mm/min. The dominant tribological phenomenon occurring during the friction was a flattening of the surface asperities as evidenced by SEM micrographs (Figure 7, Figure 8 and Figure 9). The phenomenon of flattening the surface asperities occurs both in the conditions of dry friction (Figure 7) and lubrication of sheet surface (Figure 8 and Figure 9). The flattened surfaces are separated by groves, which are remnants of the as-received surface. In the range of the analysed pressures, grooves could be observed, which also occur on the sheet surface in its as-received state. Many cracks on the surface of the sheet in its as-received state (Figure 10) have been seized (Figure 7, Figure 8 and Figure 9). In general, similar observations can be applied to sheet metals tested at a speed of 20 mm/min (Figure 11).

Lubrication is a basic and effective way to reduce the coefficient of friction and wear of mating surfaces. However, it is well-known that iron oxides, due to their low shear stress, act as a solid lubricant and reduce the coefficient of friction [54,55]. The development of smooth oxide ‘glazes’ consists of fine, crystalline oxide particles on the load-bearing areas of alloys, which can lead to a significant reduction in COF [56]. Writzl et al. [57] detected and characterized the surface oxides using confocal Raman microscopy (power 15 mW, wavelength 633 nm). The outermost layer was composed of iron oxide phases, underlain by a compound layer which consisted of oxides, nitrides, and a high-resistance martensitic layer, with accompanying carbides and nitrides provided high load capacity for the external layers, allowing them to function as solid lubricants [57]. The presence of the oxide phases is fundamental in obtaining the low COF [58]. Wang et al. [59] attributed a lubricated effect during sliding to the formation of surface oxide film containing iron oxides. As also found Brunetti et al. [55], the maintenance of the oxide layer during a sliding wear process reduces the wear rate of the system considerably.

### 3.2. ANN Analysis

The smallest value of the RMS error for the validation set and, at the same time, the largest value of Pearson’s correlation coefficient R^2^ were provided by a network with one hidden layer and nine neurons in the hidden layer (MLP 3-3:9:1-1). The most important regression statistics are presented in Table 3. The correctness of the training process is determined by the similarly high correlation value R^2^ > 0.98 for both data sets. The prognostic quality of the neural network is determined by the quotient of the standard deviation of errors and the standard deviation of the value of the dependent variable (SD Ratio):

(4) $$\mathrm{SDRatio}=\frac{\mathrm{Error}\;\mathrm{SD}}{\mathrm{Data}\;\mathrm{SD}}$$

For networks with very good forecasting capabilities, the SD ratio value should be less than 0.2. An SD ratio value greater than 1 proves that a more accurate estimation of the value of the dependent variable is its arithmetic mean determined based on the training set [60].

The purpose of the sensitivity analysis was to examine the impact of removing individual explanatory variables on the total error of the network. A sensitivity analysis was performed independently for the training set (Table 4) and the validation set (Table 5). The ‘Rank’ row in these tables lists the variables in order of importance. Oil viscosity has the greatest impact on the value of the coefficient of friction, followed by contact pressure and sliding speed. The error of the network after removing the specified variable from the dataset is given in the ‘Error’ row. A given parameter is more important the greater the increase in the error value caused by its removal. The ‘Ratio’ parameter is responsible for the quotient of the error obtained after removing the selected explanatory variable and the error obtained using the network containing all explanatory variables [61].

**Figure 12:**
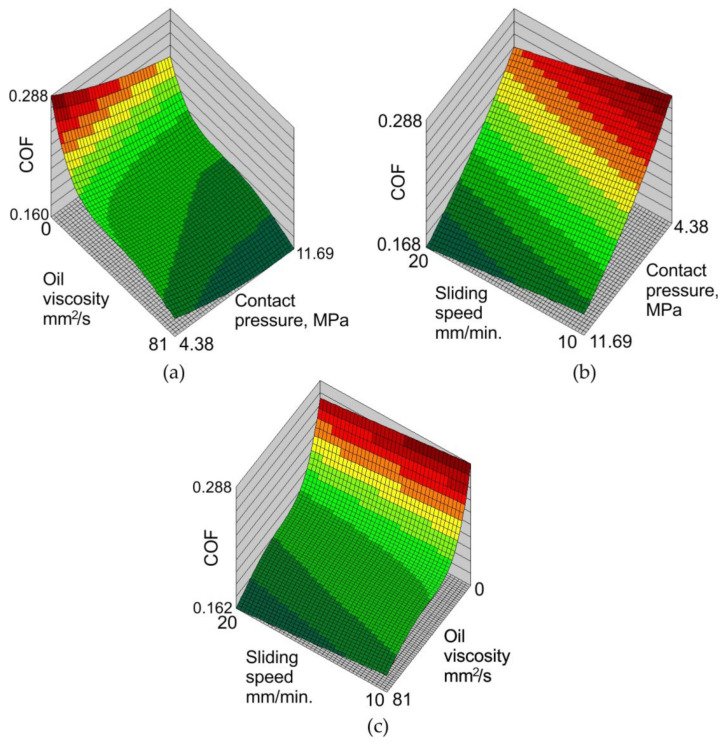
Response surfaces for the effect of (**a**) oil viscosity and contact pressure, (**b**) sliding speed and contact pressure, and (**c**) sliding speed and oil viscosity on the value of the coefficient of friction.

According to the response surfaces, the sliding speed has little effect on the change in the coefficient of friction (Figure 12b,c). As the sliding speed increases, the value of the friction coefficient decreases. Similar results were obtained by Tamai et al. [62] and Wang et al. [63], who studied the influence of the sliding speed on the coefficient of friction of galvanised steel sheets. As the sliding speed increases, the real contact area decreases, limiting the mechanical interaction of the surfaces in contact. The value of the coefficient of friction under lubrication conditions consists of two factors, the coefficient of friction of the solid, and the coefficient of friction of the lubricant [64]. Increasing the sliding speed increases the thermal effect at the summits of the asperities, causing a decrease in the viscosity of the lubricant and thus a decrease in the lubricant’s coefficient of friction. As a result, as the sliding speed increases, the coefficient of friction decreases. This effect has also been observed for dry friction conditions. At low sliding speeds, the contact surface is dominated by the elastic contact of the surface asperities, favouring the formation of the phenomenon of fluctuated slip, called the stick-slip phenomenon. A clear tendency of the COF to decrease with increased oil viscosity was observed (Figure 12a,c).

With the increase in the sliding speed, the fluctuations between the slip and friction on the contact surface decrease, with the stabilisation of conditions at higher speeds favouring a reduction in the value of the COF [65]. Increasing the viscosity of the oil reduces the COF (Figure 12a). Viscosity affects the value of the COF in combination with contact pressure. At low pressures, the high-viscosity oil is able to separate or reduce the metallic contact between the tool surface and the sheet metal. In the high-pressure range in the sheet metal formation of the relatively soft workpiece material and the hard tool, metallic contact is unavoidable, but the hydrostatic pressure of the oil is created in the closed lubricant pockets, which acts as a lubricating cushion, separating the rubbing surfaces. Therefore, a loss of lubricating properties of oils occurs faster at higher pressures.

## 4. Conclusions

This paper presents the friction test results of as-received AW-6082-T6 aluminium alloy sheets in strip draw tribological tests. The tests were carried out for lubricants commonly used in sheet metal forming with viscosity varying between 21.9 and 81 mm^2^/s. The synergistic effect of input parameters (oil viscosity, contact pressure, and sliding speed) on the COF was analysed using multilayer perceptron. Based on the results of experimental investigations, including analyses using ANNs and SEM observations, the following conclusions can be drawn:

- The lowest value of the coefficient of friction was recorded when lubricating the sheet metal surface with SAE 5W40 C3 engine oil, which is characterised as the most viscous of all tested lubricants.
- In general, the coefficient of EOL for both analysed sliding speeds was similar and varied between 16.0 and 28.9%, 19.2% and 33.8%, and 28.9 and 40.2% for LHL 32 hydraulic oil, LAN 46 machine oil, and 5W40 C3 engine oil, respectively.
- When considering the effect of contact pressure on the lubrication efficiency, it can be said that at high pressures the lubrication efficiency is lower than at low pressures. This is due to the intensification of surface flattening in high contact pressures and the reduced volume of the valleys (also known as lubricant pockets) on the sheet surface that can hold the lubricant.
- In dry friction conditions, a decreasing trend of the coefficient of friction with increasing contact pressure was observed.
- In lubricated conditions, the value of the coefficient of friction was more stable in the range of contact pressures between 4 and 9 MPa; after exceeding this value, the lubricant film was broken and a slight increase in coefficient of friction was observed.
- SEM micrographs revealed that the main friction mechanism of tested sheets in contact with the surface of the cold-work tool steel is the flattening of surface asperities.
- The trained model of MLP was characterised by high Pearson correlation (R^2^ > 0.98) for both training and validation sets. The sensitivity analysis of the input parameters on the value of coefficient of friction revealed that oil viscosity has the greatest impact on the value of the coefficient of friction, followed by contact pressure and sliding speed.

## Figures and Tables

**Table 1:** Mechanical parameters of the 6082-T6 aluminium alloy sheets.

Sample Direction	R_p0.2_, MPa	R_m_, MPa	A_80_, %	n	r	Δr
0°	314	342	13.7	0.087	0.528	−0.139
45°	307	337	14.2	0.086	0.657	
90°	313	341	12.0	0.086	0.509	

**Table 2:** Experiment design.

Test No.	Sliding Speed, mm/min	Pressure, MPa	Friction Conditions
1	10	4.38	dry friction
2	10	6.53	dry friction
3	10	8.13	dry friction
4	10	9.47	dry friction
5	10	10.63	dry friction
6	10	11.69	dry friction
7	20	4.38	dry friction
8	20	6.53	dry friction
9	20	8.13	dry friction
10	20	9.47	dry friction
11	20	10.63	dry friction
12	20	11.69	dry friction
13	10	4.38	hydraulic oil LHL 32
14	10	6.53	hydraulic oil LHL 32
15	10	8.13	hydraulic oil LHL 32
16	10	9.47	hydraulic oil LHL 32
17	10	10.63	hydraulic oil LHL 32
18	10	11.69	hydraulic oil LHL 32
19	20	4.38	hydraulic oil LHL 32
20	20	6.53	hydraulic oil LHL 32
21	20	8.13	hydraulic oil LHL 32
22	20	9.47	hydraulic oil LHL 32
23	20	10.63	hydraulic oil LHL 32
24	20	11.69	hydraulic oil LHL 32
25	10	4.38	machine oil LAN 46
26	10	6.53	machine oil LAN 46
27	10	8.13	machine oil LAN 46
28	10	9.47	machine oil LAN 46
29	10	10.63	machine oil LAN 46
30	10	11.69	machine oil LAN 46
31	20	4.38	machine oil LAN 46
32	20	6.53	machine oil LAN 46
33	20	8.13	machine oil LAN 46
34	20	9.47	machine oil LAN 46
35	20	10.63	machine oil LAN 46
36	20	11.69	machine oil LAN 46
37	10	4.38	engine oil SAE 5W-40 C3
38	10	6.53	engine oil SAE 5W-40 C3
39	10	8.13	engine oil SAE 5W-40 C3
40	10	9.47	engine oil SAE 5W-40 C3
41	10	10.63	engine oil SAE 5W-40 C3
42	10	11.69	engine oil SAE 5W-40 C3
43	20	4.38	engine oil SAE 5W-40 C3
44	20	6.53	engine oil SAE 5W-40 C3
45	20	8.13	engine oil SAE 5W-40 C3
46	20	9.47	engine oil SAE 5W-40 C3
47	20	10.63	engine oil SAE 5W-40 C3
48	20	11.69	engine oil SAE 5W-40 C3

**Table 3:** Regression statistics for MLP 3-3:9:1-1.

Parameter	Training Set	Validation Set
Data Mean	0.1974275	0.1870625
Data SD	0.03705	0.03334
Error Mean	8.89 × 10^−5^	−0.0006155
Error SD	0.005955	0.005251
Abs E. Mean	0.005177	0.003623
SD Ratio	0.1607267	0.157522
Correlation	0.9869992	0.9876078

**Table 4:** Sensitivity analysis for the training set.

Parameter	Sliding Speed	Oil Viscosity	Contact Pressure
Rank	3	1	2
Error	0.008	0.037	0.011
Ratio	1.372	6.363	1.978

**Table 5:** Sensitivity analysis for the validation set.

Parameter	Sliding Speed	Oil Viscosity	Contact Pressure
Rank	3	1	2
Error	0.009	0.028	0.010
Ratio	1.902	5.800	2.032

## Data Availability

Data is contained within the article.
